# Performance of ‘Gala Select’ and ‘Fuji Suprema’ grafted on Geneva series rootstocks under fallow land and replanting conditions in southern Brazil

**DOI:** 10.1016/j.heliyon.2023.e22125

**Published:** 2023-11-10

**Authors:** Pricila Santos da Silva, Juliana Martins de Lima, Marllon Fernando Soares dos Santos, Daiana Petry, Leo Rufato, Francine Regianini Nerbass, Amauri Bogo

**Affiliations:** Crop Production Graduate Program, Santa Catarina State University-UDESC, Lages, SC, Brazil

**Keywords:** Malus domestica Borkh., Productive efficiency, Vigor control, Fruit production and quality, Yield

## Abstract

**Background:**

Rootstocks less vigorous are among the most crucial management techniques to modernize fruit cultivation. Replanting with fallow land has become necessary due to a lack of land to establish new orchards.

**Objective:**

The aim of this study is to determine the effect of various rootstocks of the American Geneva® series on the yield performance of the apple (*Malus domestica* Borkh) cultivars ‘Gala Select’ and ‘Fuji Suprema’ under replanting conditions in southern Brazil. *Methods*: After two years of fallow land, the experiments were initiated in 2017 and conducted during the 2018, 2019, 2020, and 2021 growing seasons in Painel and Caxias do Sul municipalities at the Santa Catarina and the Rio Grande do Sul State, respectively. The ‘Gala Select’ and ‘Fuji Suprema’ were grafted onto the G.202, G.814, G.210, and G.213 Geneva series rootstocks in a tall spindle training system using a randomized complete block design with four replicates. Principal component analysis (PCA) was used to assess the interrelationship among the variable's vigor, productivity, and fruit quality.

**Results:**

The PCA result showed significant differences in vigor, productivity, and fruit quality when the G.210 and G.213 and G.814 and G.213 Geneva series rootstocks were used in combination with Gala Select and Fuji Suprema cultivars, respectively. The PCA analysis clustered all cultivar/rootstock combinations into two groups, based on their vigor and productivity and the yield performance and fruit quality data, that differed significantly among combinations and regions. The ‘Gala Select’/G.202 and ‘Fuji Suprema’/G.213 combinations were less vigorous than the ‘Gala Select’/G.210 and ‘Fuji Suprema’/G.814 combinations. However, ‘Gala Select’/G.210 (semi-dwarfing) and G.213 (dwarfing) are the combinations with high yield performance, productive efficiency and fruit quality, being more reliable to the producer, and less vigorous, resulting in lower labor costs under replating conditions, with two years of fallow land, from 2018 until 2021 growing seasons.

## 1 Introduction

Apples (*Malus domestica* Borkh.) are the second most produced and third most consumed temperate fruit in Brazil. The states of Santa Catarina and the Rio Grande do Sul States are the leading producers, with 33 thousand hectares of production area [1]. The demand for new cultivars, clones, rootstocks, and management technologies by Brazilian apple growers is the primary factor driving the expansion of apple production [6,7]. However, the average yield of the Brazilian apple crop in 2019 was estimated to be around 37.7 t ha^−1^ [12]. This is a result of the use of vigorous rootstocks and low plant densities [17].

The Brazilian apple orchards longevity seldom exceeds 25–30 years due to the frequent replacement of scion cultivars and new planting density technologies that require the replacement of vigorous rootstocks by the most dwarfing ones [19]. Therefore, because of the low availability of new areas in the most apple productive States of southern Brazil, new orchards need to establish on replanting soils and areas. This activity, can seriously compromise the apple tree development and yield performance when new orchards are planted on areas previously cultivated with temperate-zone fruit trees [14]. The issue of replanting plants can lead to growth suppression and reduced productivity of trees planted in older orchards, potentially rendering the activity unprofitable. The exact etiology of replanting plant problems is unknown and their nature is complex due to the presence of different biotic and abiotic factors. The correct rootstock selection is more beneficial than soil fumigation in controlling the problem of apple replanting associated with the problem [27].

It has been extremely difficult to find new land for the establishment of new orchards, making it necessary to reuse orchard land. Any attempt to cultivate a new orchard with high quality and high yields in an unproven area increases the grower's risk. Most of the land available for establishing new orchards is designated as a replanting area and the soil fumigation for controlling replant problems is not permitted in Brazil. According to Denardi [5], apple plant performance may vary based on the used rootstock and replanting soil conditions. According to Reig [21], it is likely that the rootstock-induced changes in the concentrations of mineral nutrients can have subtle or dramatic effects on the physiological machinery of the tree, resulting in changes in fruit production and quality. In general, vigorous rootstocks are more resistant to apple replanting disease than dwarfing rootstocks, and tolerance of their root system to harmful agents appears to play a crucial role. The use of dwarfing rootstocks to increase the planting density has been the most significant change in apple production [23]. The primary benefits of increasing plant density are increased productivity [19], precocious production, high fruit quality, and low labor costs [11]. However, it is important to select the proper rootstocks for the effective management of plant sizes [16].

The first apple orchards planted in the southern Brazil on replanting soils were grafted on the MM.111, MI.793, and M.25 British rootstocks [24], followed by M.7 durring the 1990s [5], and more recently by Marubakaido rootstock and the M.9 interstem [4,5]. Despite of these rootstocks has good performance, most of them show unsatisfactory tolerance to “apple replant disease” (ARD) and insect and rot problems [24].

The apple crop breeding program of Cornell University/USA has been developing new American Geneva series rootstocks (CG series). Some of these rootstocks, specially, the G.202, G.814, G.210, and G.213 rootstocks were selected due to resistance to collar root rot and to woolly apple aphid-lanigerus [[8], [9], [10], [11], [12], [13], [14], [15], [16], [17], [18], [19], [20], [21], [22], [23], [24]], adequate tolerance to apple replant disease [ [7,8]], increased productivity, ability to induce better canopy budding [15], and a better angle of branch insertion to the stem [7]. The climate and environmental conditions of the local region are fundamental factors in the selection of a suitable rootstock for a cultivar [28]. All of these CG series rootstock features are required and useful for southern Brazil's environmental conditions [15].

Rufato [24] evaluated different rootstocks, with a particular focus on the G.213 rootstock. This rootstock requires only a few chilling hours and budding degree days, and it also demonstrates resistance to diseases and pests. The study revealed that ‘Maxi Gala’ apple trees grafted onto G.213, G.202, G.757, G.814, M.9, and CAT16 rootstocks exhibited higher concentrations of jasmonic acid and *trans*-zeatin-riboside, along with lower concentrations of abscisic acid (ABA). The decreased ABA concentration and increased *trans*-zeatin-riboside concentration account for the easier budding process, requiring fewer chilling hours. Jasmonic acid is acknowledged as a stress hormone that plants produce in response to both biotic and abiotic stresses, and can trigger certain levels of plant resistance. The authors associated the elevated concentration of jasmonic acid with a potential mechanism for disease resistance in the specific replanting areas examined in this study. However, additional research on the development of CG series rootstocks in various apple-growing regions of southern Brazil is still necessary, particularly in replanting conditions with fallow land. Accordingly, the objective of the current study was to determine the effect of various Geneva series rootstocks on the yield performance of ‘Gala Select’ and ‘Fuji Suprema’ under replanting conditions with two years of fallow land in the edaphoclimatic conditions of southern Brazil during 2018/19, 2019/20, and 2020/21 growing seasons.

## 2 Materials and methods

The experiments were carried out in commercial apple orchards in replanting conditions with two years of fallow land in Painel/SC and Caxias do Sul/RS Municipalities, Santa Catarina (SC) and the Rio Grande do Sul (RS) states, respectively, during 2018/19, 2019/20, and 2020/21 growing seasons. The site of the experiments was located at 29°12′ 94″ S, 51°15′83″ W at an altitude of 830 m above sea level in Caxias do Sul/RS. The climate of Painel/SC and Caxias do Sul/RS are Cfb, humid mesothermal with mild summers and Cfa, humid subtropical with hot summers and cold and rainy winters, according to Köppen's classification [18]. Daily rainfall, relative humidity, hourly temperatures, and chilling hours (minimum period in hours of cold weather after which a fruit-bearing tree will blossom) were recorded at Pinheiros Altos (Painel/SC) and Basf/SA (Caxias do Sul/RS). In both regions, the weather station (Davis Pro2-6153; Onset Computer Corp., Pocasset, MA, USA) was situated approximately 20 m away from the orchards.

The apple orchards were planted in 2017 in a replanting condition with two years of fallow land in the Painel/SC and Caxias do Sul/RS regions. The soil of the Painel/SC region fall into the classes cambisol humic, neossol litolic, and nitossol haplic, formed from riodacite rock and basalt, and the Caxias do Sul/RS region into the latossolo bruno dystrophic, with smooth to wavy relief, containing high levels of clay and aluminum, according to the Brazilian Soil Classification System [26]. Soil analysis before the start of the experiment revealed the following average composition for Painel/SC and Caxias do Sul/RS regions at the 20–40 cm depths: 3.2 mg kg^−1^ P; 0.21 meq 100 L^−1^ K; 13 meq 100 L^−1^ Ca; 6.6 meq 100 L^−1^ Mg; 5.1 % organic matter; and 0.11 % Al. In order to correct the acidity, liming was carried out in soil, in order to raise the soil pH in water to 6.0. In the correction fertilization, K_2_O and P_2_O_5_ were applied when necessary, following the recommendation of the Soil Chemistry and Fertility Committee RS/SC [3]. ‘Gala Select’ and ‘Fuji Suprema’ seedling were grafted 10 cm above the ground (whip and tongue grafting) on the G.202, G.814, G.210, and G.213 rootstocks and trained to the tall spindle at a high density. The G.202, G.814, G.210, and G.213 rootstocks originate from the Cornell University Breeding Program. They were chosen for their resistance to collar root rot and woolly apple aphid, and some of them exhibit suitable tolerance to Apple Replant Disease (ARD). The selection of ‘Gala Select’ and ‘Fuji Suprema’ was based on their widespread utilization as prominent clones in southern Brazil.

Trees in the orchard were planted in Painel/SC with a spacing of 3.5 m × 0.90 m and a plant density of 3174 plants ha^−1^ and 3.5 m × 1.10 m, with a plant density of 2597 plants ha^−1^. Similarly, in Caxias do Sul/RS, trees were planted with a spacing of 3.5 m × 0.90 m and a plant density of 3174 plants ha^−1^ and 3.5 m × 1.0 m and a plant density of 2857 plants ha^−1^ for ‘Gala Select’ and ‘Fuji Suprema’, respectively. The orchards in the Painel/SC and Caxias do Sul/RS municipalities were established using a drip irrigation system right from planting, and they were protected with anti-hail nets. Pruning activities were conducted to eliminate branches that could compete with the central leader. Additionally, chemical fruit thinning and integrated pest management practices were implemented for both regions and cultivars, according to local recommendation.

Yield performance of ‘Gala Select’ and ‘Fuji Suprema’ on Geneva series rootstocks was evaluated using the following variables:

• Total plant height (m), measured with a flexible tape graduated in millimeters, from the grafting point to the apex of the plant;
• Canopy volume (m^3^): obtained by measuring L = canopy length, H = canopy height from first branch insertion point to apex, W = canopy width, and using equation (L × H × W) [15];
• Trunk cross-sectional area (TCSA) (cm^2^), obtained by the average of the longitudinal and transversal measure of trunk diameter at 10 cm above the grafting point. To convert truck diameters to TCSA, equation A = (πd2)/4 was used, where d = trunk diameter;
• Number of branches, obtained by counting all branches larger than 10 cm extending from the central leader;
• Productive efficiency, calculated through the ratio of fruit weight mean per plant (kg·cm^2^) to crown trunk cross-sectional area (cm^2^), expressed in kg of fruits produced per square centimeter of TCSA;
• Cumulative yield (t·ha^−1^), obtained by adding all crop yields from 2018 to 2021;
• Inc t·cm^2^ (kg): the average increment of production per increment of TCSA.

The variables total plant height, canopy volume, TCSA, and number of branches were measured during winter time between the beginning of June and the end of July for each growing season. The ‘Gala Select’ and ‘Fuji Suprema’ combinations harvest began approximately 120 (February) and 175 days (March) after flowering, respectively, of each growing season when the fruits have reached commercial maturity as determined by the starch-iodine index (4–5), pulp fruit firmness (80–90 N), and soluble solids (11–12° Brix).

The fruits were counted and weighed and subsequently, the fruit quality of ‘Gala Select’ and ‘Fuji Suprema’ on Geneva series rootstocks were determined by the following variables:

• Category classification (%), where a sample of 20 fruits per plot was used for CAT1 and CAT2 classification. CAT1 fruits had a red color of ≥50 % and CAT2 had a red color of <50 %. Other defects were not accounted for in this classification;
• Fruit size (%), a sample of 20 fruits per plot was used to classify the fruits size. A flexible ruler graduated in cm was used physically to measure the fruits diameter. The fruit classification was made by fruit caliber category, measured through the proportional representation of fruits in each caliber category. The numbering of 300, 250, 220, 198, 180, 165, 150, 135, 120, 110, 100, 90, 80, 70, and 60, representing the fruits number fitted inside per 18 kg box. Fruits category between 100 and 120 was classified as large fruits (caliber 100–120), between 120 and 150 as medium fruits (caliber >120–150), and bigger than 150 as small fruits (caliber >150).
• Starch-iodine index (scale 1 to 9), obtained by reacting fruit starch with a solution of 12 g of metallic iodine and 24 g of potassium iodide in 1 L of distilled water. After classifying the fruits on a scale from 1 to 9, they were categorized as follows: 1–3: immature fruits, 4–6: ripe fruits, and 7–9: overripe fruits;
• Firmness (N), where 50 fruits were measured per plot using a digital texture analyzer with an 11 mm tip. The measurement was performed in the equatorial zone of the fruit after two epidermis discs of about 1 cm in diameter were removed from opposite sides;
• Total soluble solids (TSS) (°Brix), measured with a digital refractometer from the juice extracted from a slice of each fruit in a sample of 50 fruits.

The experiments were conducted in a completely randomized experimental block of 10 plants along each row with four replications. The yield performance and fruit quality were analyzed for each Region/Municipality and the results were submitted to the Shapiro-Wilk and Bartlett tests. The data were transformed into √(*x* + 0.5) when necessary. Using R software [20], significance tests were conducted using analysis of variance, and the means were grouped using the Tukey test at 5 %. Multivariate analysis was performed using principal component analysis (PCA).

The PCA is an unsupervised learning algorithm technique that allows to summarize and to visualize the information in a data set containing observations described by multiple inter-correlated quantitative variables. Thus, the PCA is used to extract the important information from a multivariate data table and to express this information as a set of few new variables called principal components. These new variables correspond to a linear combination of the originals. Correlation analyses and ANOVA were employed as supplementary analytical techniques, utilized selectively to provide additional support and interpretation for the PCA results.

## 3 Result

**Fig. 1:**
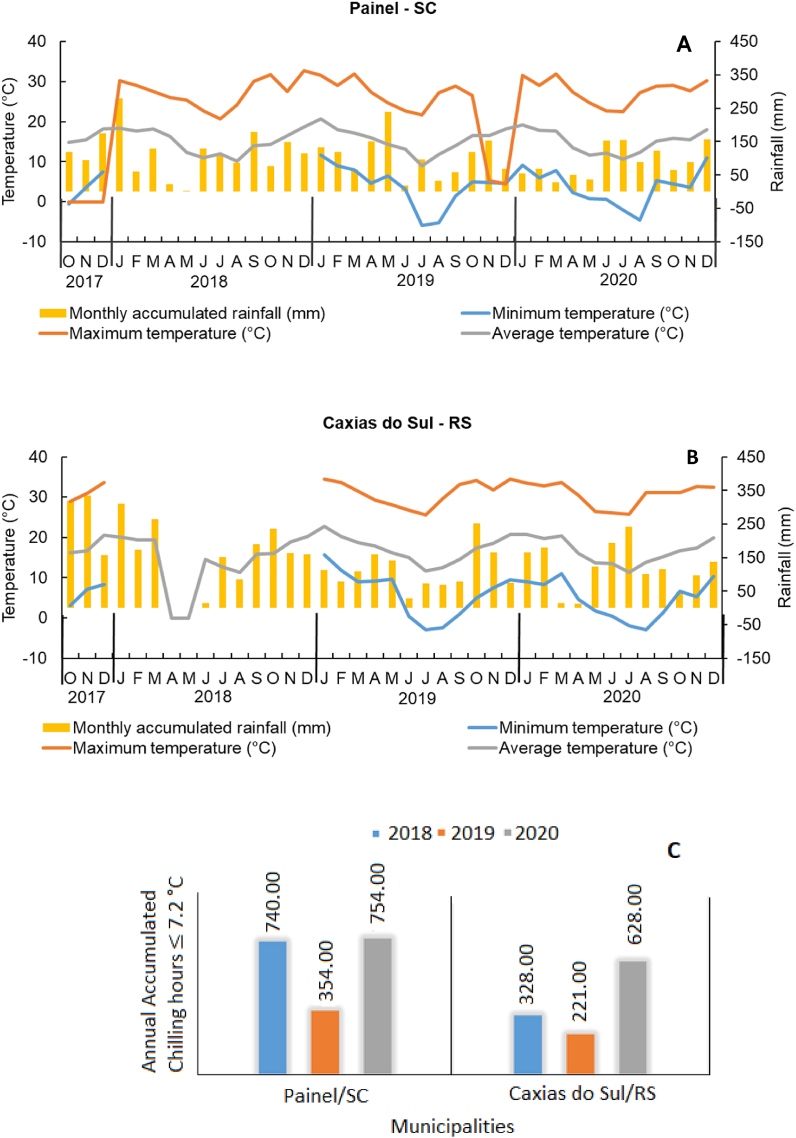
Average monthly accumulated rainfall (mm), maximum, average and minimum temperature (°C) at Painel/SC (A) and Caxias do Sul/RS (B) and Annual accumulated chilling hours ≤7,2 °C (C) municipality, southern Brazil, from October 2017 to December 2020, across the three growing seasons.

**Fig. 2:**
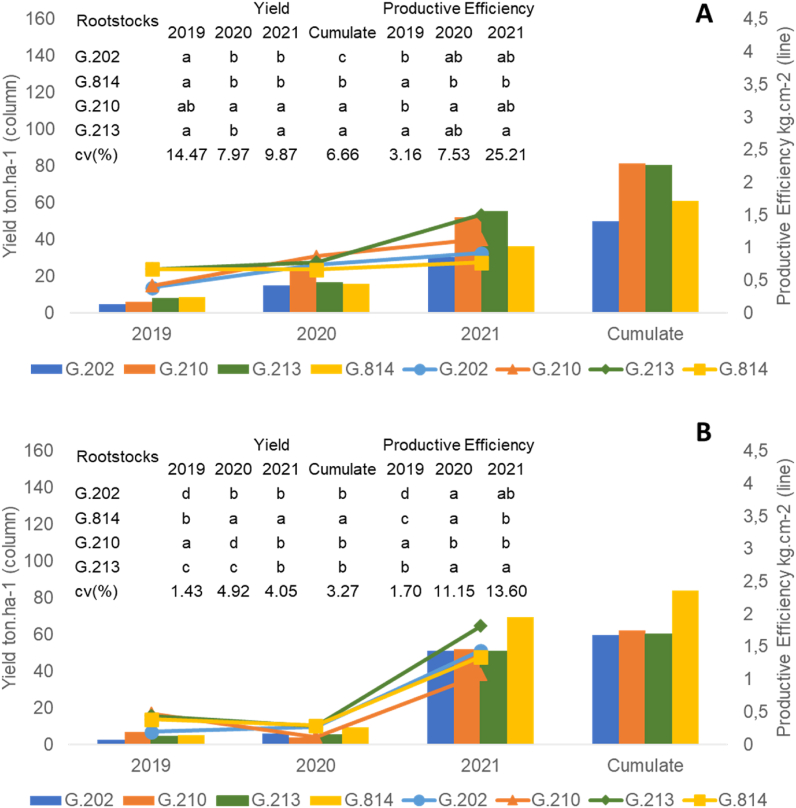
Yield and productive efficiency of ‘Gala Select’ (A) and ‘Fuji Suprema’ (B) grafted on a series Geneva® rootstocks, under replanting area at Painel/SC municipality, southern Brazil.

During the evaluation period from October 2017 to December 2020, the average maximum and minimum temperatures, total rainfall, and chilling hours were 19.7 °C, 10.3 °C, 1654 mm, and 630 h at the Panel/SC and 21.9 °C, 12.6 °C, 1417 mm, and 392 h at the Caxias do Sul/RS (Fig. 2), respectively (Fig. 1).

The average minimum and maximum temperatures during the phenological stage of beginning of sprouting (August), fruit growth and development (December to January), and harvest (February to April) were 10°C-12 °C, 18°C-20 °C, and 17°C-20 °C for the region of Panel/SC and 12°C-13 °C, 20°C-22 °C, and 19°C-21 °C for the region of Caxias do Sul/RS, respectively (Fig. 1, Fig. 2A). However, the average maximum temperatures from the beginning (February) to the end of the harvest (April) was approximately 30 °C and 32 °C for Panel/SC and Caxias do Sul/RS, respectively (Fig. 1, Fig. 2A). Additionally, the number of chilling hours from beginning of Autumn (April) to end of Winter (September) was 740, 354, and 754 h, in Panel/SC; while in Caxias do Sul/RS it was 328, 221, and 628 h during 2018, 2019, and 2020 growing seasons, respectively (Fig. 1C). According to Csihon [2], the significance of conducting cultivar testing across diverse climatic conditions lies in the substantial influence these test outcomes wield over growers' selection of cultivars for establishing new plantations. Despite sharing a similar soil classification, the two regions experience distinct climatic conditions. In Painel/SC, a higher number of chilling hours and lower minimum and maximum temperatures were observed compared to Caxias do Sul/RS (Fig. 1).

**Fig. 3:**
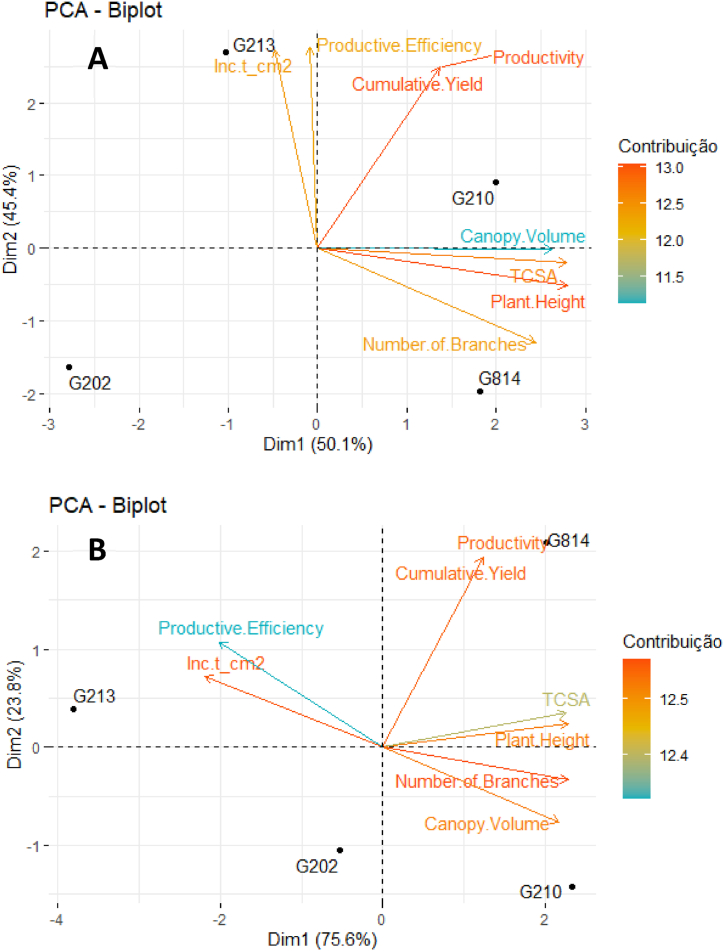
Vigor-productive efficiency relationship of Gala Select (A) and Fuji Suprema (B) cultivars grafted on a series of Geneva® rootstocks, under replanting area at Painel municipality, southern Brazil. According to ‘Gala Select’, the first component separated the rootstocks based on their vigor, while the second component separated them based on the yield performance that they provided to canopy cultivar. According to ‘Fuji Suprema’, the first principal component variables that represent the vigor were inversely proportional to the productive efficiency and proportional to the yield performance.

**Fig. 4:**
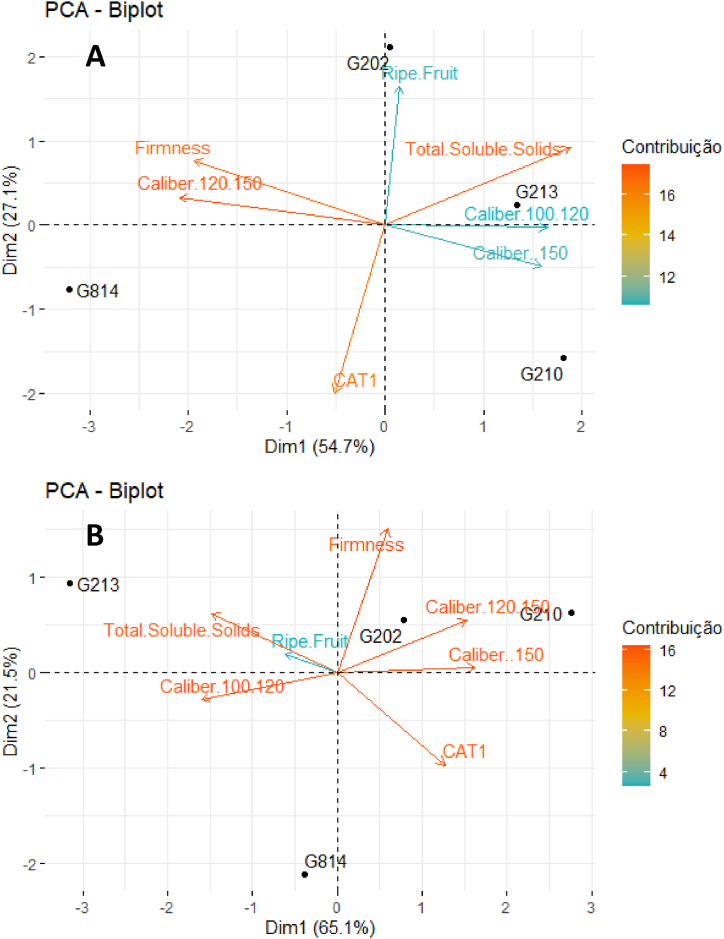
Fruit quality analysis of the cultivar Gala Select (A) and Fuji Suprema (B) grafted on a series of Geneva® rootstocks, under replanting area at Painel municipality, southern Brazil. According to ‘Gala Select’, the first main component separated the rootstocks mainly by fruits caliber and firmness. Medium caliber and firmness were inversely proportional to smaller and larger calibers. The second main component differentiated the fruit quality mainly by the fruits category. According to ‘Fuji Suprema’, the first main component correlated the CAT1 category with the largest and smallest fruit caliber, and the second main component differentiated the rootstocks mainly by fruits firmness.

**Fig. 5:**
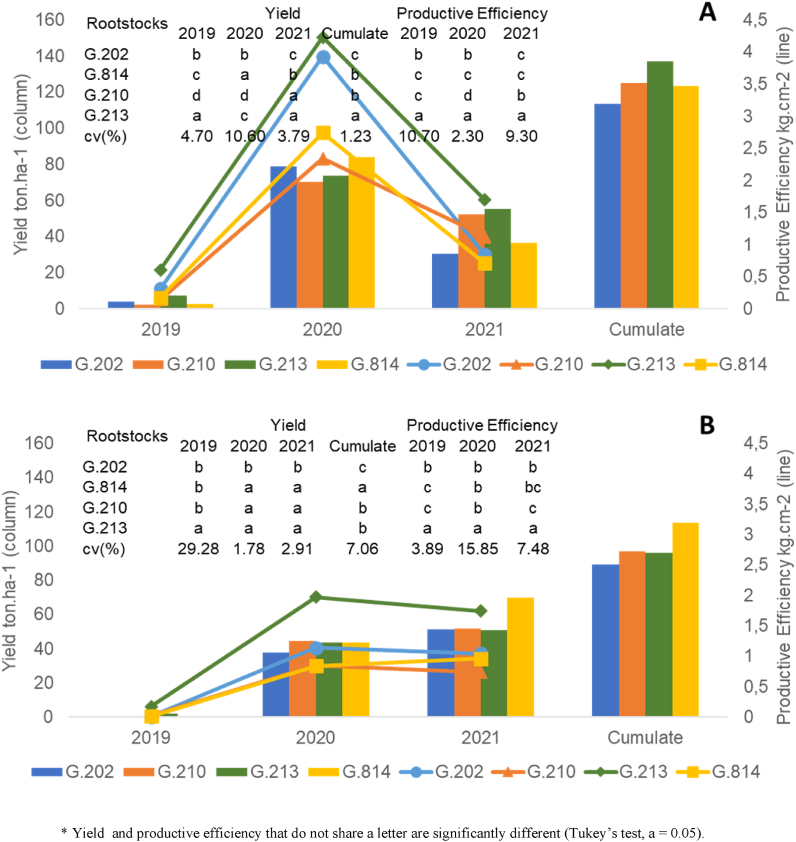
Yield and productive efficiency of the ‘Gala Select’ (A) and ‘Fuji Suprema’ (B) grafted on a series of Geneva® rootstocks, under replanting area in Caxias do Sul municipality, southern Brazil.

**Fig. 6:**
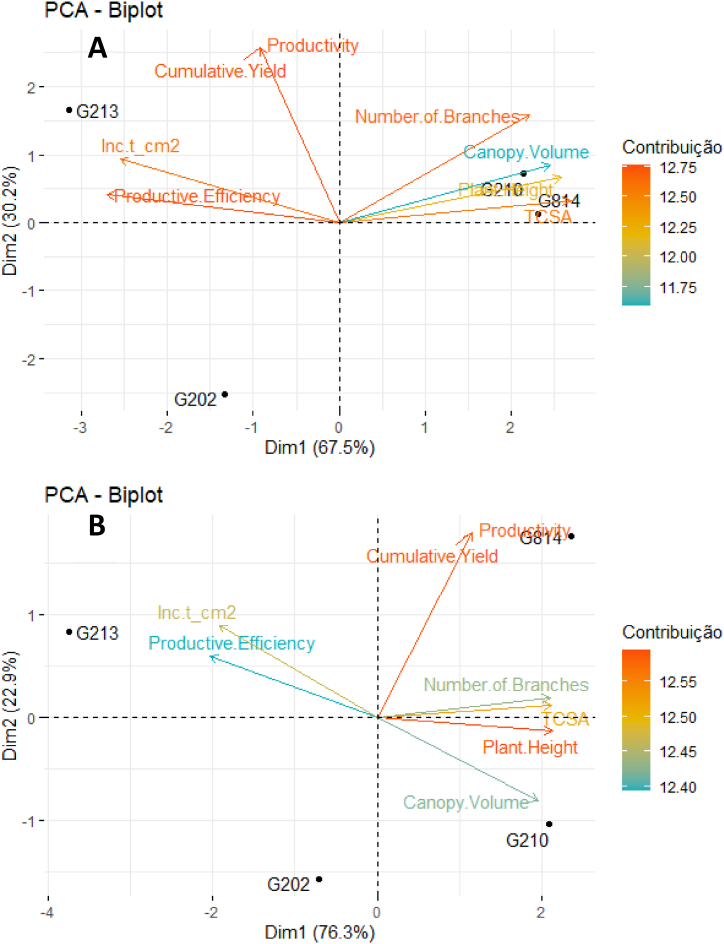
Vigor-productive efficiency relationship of the ‘Gala Select’ (A) and ‘Fuji Suprema’ (B) on the Geneva® series rootstocks, under replanting area in Caxias do Sul municipality, southern Brazil. For both Gala Select and Fuji Suprema, the first component separated the rootstocks based on their vigor, inversely proportional to productive efficiency, while the second component separated them based on the yield performance that they provided to the canopy cultivars.

**Fig. 7:**
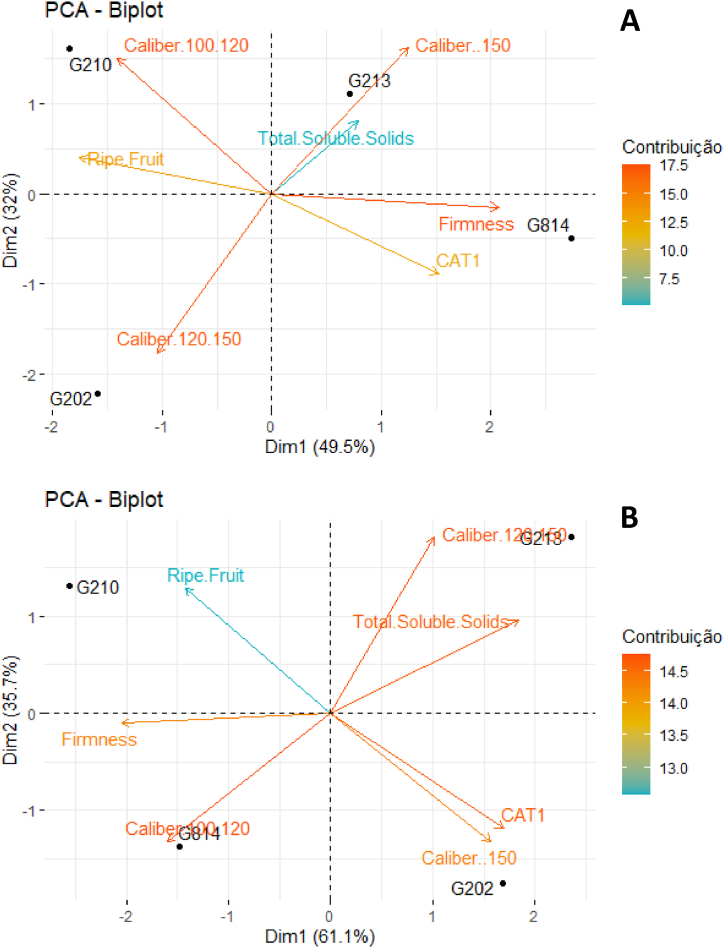
Fruit quality analysis of the ‘Gala Select’ (A) and ‘Fuji Suprema’ (B) grafted on a series of Geneva® rootstocks, under replanting area in Caxias do Sul municipality, southern Brazil. According to ‘Gala Select’, the first main component separated the rootstocks mainly by the fruits caliber that were inversely proportional to fruits firmness and category. The second main component differentiated the rootstocks mainly by the fruits medium caliber that were inversely proportional to the fruits smaller caliber. According to ‘Fuji Suprema’, the first main component differentiated the rootstocks mainly by the fruits firmness that were correlated with fruits larger calibers that were inversely proportional of fruits average calibers and soluble solids content. The second main component differentiated the rootstocks mainly by fruits category.

Fig. 2, Fig. 3, Fig. 4, Fig. 5, Fig. 6, Fig. 7 depict the yield-productive efficiency, vigor-productive efficiency and fruit quality results of ‘Gala Select’ and ‘Fuji Suprema’ grafted on a series Geneva® rootstocks over the 2018/19, 2019/20 and 2020/21 growing seasons, respectively. The comparison between cultivars was not performed on the basis of specific cultivars characteristics, and the rootstocks were compared within each cultivar.

**Fig. 8:**
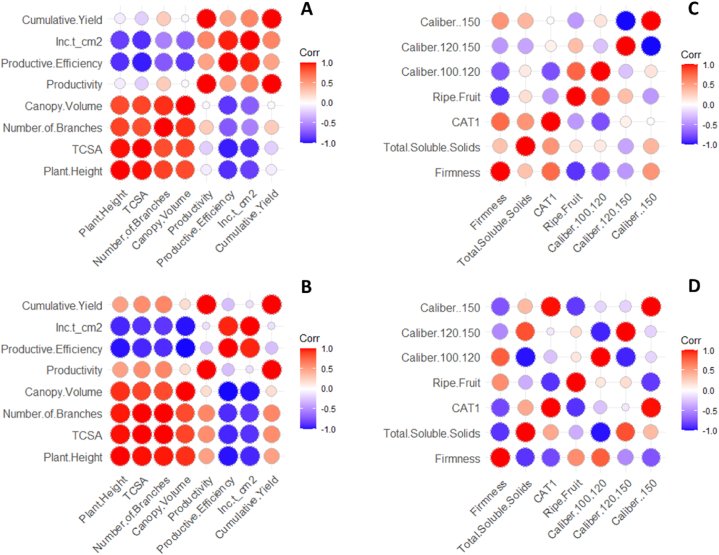
Correlation of vigor-productive efficiency variables of the ‘Gala Select’ (A) and ‘Fuji Suprema’ (B), and Fruit quality variables of the ‘Gala Select’ (A) and ‘Fuji Suprema’ (B) grafted on a series of Geneva® rootstocks, under replanting area in Painel municipality, southern Brazil.

**Fig. 9:**
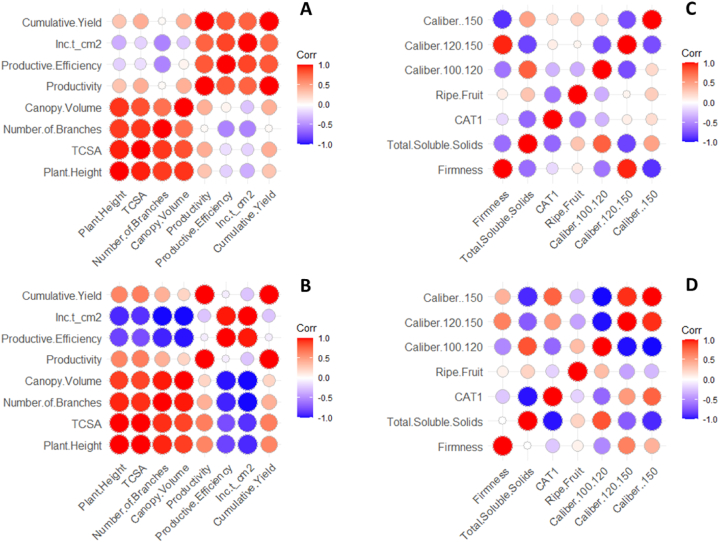
Correlation of vigor-productive efficiency variables of the ‘Gala Select’ (A) and ‘Fuji Suprema’ (B), and Fruit quality variables of the ‘Gala Select’ (A) and ‘Fuji Suprema’ (B) grafted on a series of Geneva® rootstocks, under replanting area in Caxias do Sul municipality, southern Brazil.

The yield performance and fruit quality data of ‘Gala Select’ and ‘Fuji Suprema’ grafted on Geneva series rootstocks differed significantly. In addition, the principal component analysis (PCA) data showed the best variables that can describe the effect of the G.814, G.210, G.202, and G.213 rootstocks on ‘Gala Select’ and ‘Fuji Suprema’ yield performance and fruit quality in different climatic conditions of the Painel/SC and Caxias do Sul/RS (Fig. 3, Fig. 4, Fig. 6, Fig. 7). To further enhance and reinforce the interpretations of the PCA, Fig. 8, Fig. 9 depict the correlation matrices between variables for the Painel/SC and Caxias do Sul/RS municipalities, respectively.

### 3.1 Painel/SC experimental area

Fig. 3 depict the PCA result of the yield performance of the ‘Gala Select’ and ‘Fuji Suprema’ cultivars grafted on the G.814, G.210, G.202, and G.213 rootstocks. The points represent the rootstocks, and the axes represent the vectors that describe the variable weights used to depict the behavior of the first two major components. Table 1, Table 2 display the means of the measured variables, aiming to provide support for the interpretations of the PCA. For both cultivars the first component separated the rootstocks based on their vigor, while the second component separated them based on the yield performance that they provided to the canopy cultivars. The distance between the G.202 and G.213 and the G.210 and G.814 rootstocks groups represented the vigor, because the G.202 and G.213 and the G.210 and G.814 are dwarf and semi-dwarf rootstocks, respectively. Despite these two vigor groups of rootstocks, the yield performance of ‘Gala Select’ was unrelated to vigor, with the semi-dwarf G.210 (82 t ha^−1^) and dwarf G.213 (80 t ha^−1^) rootstocks producing the highest yields. As evidenced by the second principal component, the productivity of the rootstocks G.213 and G.210 are related to the greater productive efficiency of both rootstocks. The ‘Gala Select’/G.202 combination demonstrated a correlation between vigor and yield performance, with the lowest yield performance (45 t ha^−1^) and productive efficiency (2.0 kg cm^−2^).

**Table 1:** Vigor-productive variables (mean) of the ‘Gala Select’ (A) and ‘Fuji Suprema’ (B) on the Geneva® series rootstocks, under replanting area in Painel municipality, southern Brazil.

Cultivar	Rootstock	Plant Height	TCSA	Number of Branches	Canopy Volume	Productivity	Cumulative Yield	Productive Efficiency	Inc t_cm2
**Painel Gala**	G.202	2.67	7.18	18.15	1.01	16.69	50.06	0.68	3.43
	G.210	2.98	9.25	21.07	1.73	27.14	81.42	0.80	4.56
	G.213	2.75	7.56	17.03	1.52	26.86	80.57	0.98	5.27
	G.814	3.02	8.89	21.19	2.05	20.37	61.11	0.70	2.43
**Painel Fuji**	G.202	3.04	9.10	21.78	2.44	19.87	59.61	0.64	4.35
	G.210	3.29	12.24	25.05	3.08	20.75	62.26	0.56	3.36
	G.213	2.83	7.17	17.24	1.78	20.03	60.09	0.85	5.91
	G.814	3.30	12.49	23.70	2.59	27.98	83.93	0.67	4.15

**Table 2:** Qualitative variables (mean) of the ‘Gala Select’ (A) and ‘Fuji Suprema’ (B) on the Geneva® series rootstocks, under replanting area in Painel municipality, southern Brazil.

Cultivar	Rootstock	Firmness	Total Soluble Solids	CAT1	Ripe Fruit	Caliber 100-120	Caliber 120-150	Caliber >150
**Painel Gala**	G.202	89.45	12.98	76.25	95.00	3.75	57.50	38.75
	G.210	85.90	12.81	91.88	67.50	5.00	53.75	41.25
	G.213	88.19	13.14	80.00	60.00	7.50	55.00	37.50
	G.814	91.30	11.94	90.63	62.50	2.50	61.25	36.25
**Painel Fuji**	G.202	77.77	14.19	98.75	50.00	33.75	43.75	22.50
	G.210	78.51	13.60	100.00	77.50	26.25	46.25	27.50
	G.213	77.60	14.84	97.50	85.00	47.96	36.78	15.26
	G.814	75.55	13.90	100.00	70.00	41.12	38.42	20.46

The first principal component of ‘Fuji Suprema’ (75.6 % of the data variation) showed an inversely proportional relationship of vigor and productive efficiency and a proportional relationship of vigor and productivity. Thus, the G.213 rootstock presented higher productive efficiency, but it was not the rootstock with the highest productivity. The G.814 rootstock was the most productive when compared with all other vigorous rootstocks grafted on “Fuji Suprema’ (Fig. 3).

The fact that the ‘Gala Select’ and ‘Fuji Suprema’ “inc t cm-2” variable was strongly positively correlated with the G.213 rootstock, as depicted in Fig. 3, indicates that the yield increment per TCSA unit was substantial for the G.213 rootstock. This feature indicates a constant growth in the yield performance of both cultivars under G.213 rootstock, even with a small increase in the cross-sectional area of the trunk.

There was a significant difference (p ≤ 0.05) in the cumulative yield performance and productive efficiency of ‘Gala Select’ and ‘Fuji Suprema’ grafted on Geneva series rootstocks. Yield performance and productive efficiency variables did not distinguish dwarf (G.213) and semi-dwarf (G.210) rootstocks for ‘Gala Select’ and ‘Fuji Suprema’ (Fig. 3A and B). The ‘Gala Select’/G.210 combination exhibited the highest cumulative yield (82 t ha^−1^) and was 2.5 %, 34.5 %, and 85 % more productive than ‘Gala Select’/G.213 (80 t ha^−1^), ‘Gala Select’/G.814 (61 t ha^−1^), and ‘Gala Select’/G.202 (45 t ha^−1^) in replanting conditions with two years of fallow land, during 2018/19, 2019/20, and 2020/21 growing seasons, respectively (Fig. 2A). However, the ‘Gala Select’/G.213 combination demonstrated the highest productive efficiency (2.9 kg cm^−2^) despite being only 20.8 % greater than ‘Gala Select’/G.210 (2.4 kg cm^−2^) (Fig. 2A).

‘Fuji Suprema’/G814 combination had the highest yield performance (83 t ha^−1^), followed by ‘Fuji Suprema’/G.210 (61 t ha^−1^), ‘Fuji Suprema’/G.213 (60 t ha^−1^), and ‘Fuji Suprema’/G.202 (59 t ha^−1^) (Fig. 2B). ‘Fuji Suprema’/G.814 (83 t ha^−1^) combination was 39 % more productive than ‘Fuji Suprema’/G.202 over the course of three years (59 t ha^−1^). However, the ‘Fuji Suprema’/G.213 combination demonstrated the highest productive efficiency at 2.5 kg cm^−2^ (Fig. 2B).

The fruit size was the primary factor that distinguished the impact of the rootstocks on fruit quality. The G.210 and G.213 rootstocks were distinguished primarily by the vector of largest caliber of fruit for the ‘Gala Select’ (Fig. 4A). The ‘Gala Select’/G.210 and ‘Gala Select’/G.213 combinations produced the plants with the highest fruit yield and caliber fruits (Fig. 4A). These two combinations ‘Gala Select’/G.210 and ‘Gala Select’/G.213 had the highest yield performance at 82 t ha^−1^ and 80 t ha^−1^, respectively, and produced fruits with a larger fruit caliber. The performance of the ‘Fuji Suprema’/G.210 and ‘Fuji Suprema’/G.814 combinations was identical (Fig. 4B). At the time of harvest, the ‘Gala Select’/G.213 and ‘Fuji Suprema’/G.213 combinations showed the highest soluble solids content and starch-iodine index (more ripe fruits) (Fig. 4B).

### 3.2 Caxias do Sul/RS experimental area

Fig. 6A and B depict the PCA results of the vigor-productive efficiency relationship of the ‘Gala Select’ and ‘Fuji Suprema’ on the Geneva® series rootstocks, under replanting area in Caxias do Sul municipality, southern Brazil. The points represent the rootstocks, while the axes represent the vectors that describe the weight of the various yield performance variables in order to depict the behavior of the first two major components. Table 3, Table 4 display the means of the measured variables, aiming to provide support for the interpretations of the PCA. According to vigor, there was a constant separation among ‘Gala Select’ and ‘Fuji Suprema’/G.210, ‘Gala Select’ and ‘Fuji Suprema’/G.814, ‘Gala Select’ and ‘Fuji Suprema’/G.213, and ‘Gala Select’ and ‘Fuji Suprema’/G.202. ‘Gala Select’ and ‘Fuji Suprema’ were less vigorous when grown on dwarfing rootstocks than on semi-dwarfing rootstocks. The productive efficiency of the combinations ‘Gala Select’ and ‘Fuji Suprema’/G.213 was 73 % and 110 % greater than that of the combinations ‘Gala Select’ and ‘Fuji Suprema’/G.210, respectively. ‘Gala Select’ and ‘Fuji Suprema’/G.202 were the least productive combinations (Fig. 6A and B), and despite the fact that ‘Fuji Suprema’/G.210 and ‘Fuji Suprema’/G.814 were combinations with semi-dwarfing rootstocks, their yield performance was superior to that of combinations with dwarfing rootstocks. The greater vigor induced by the G.814 rootstock in the ‘Fuji Suprema’ had no effect on the cumulative yield performance (115 t ha^−1^) that was 34 % greater than when grafted on the G.202 rootstock throughout all growth seasons (86 t ha^−1^).

**Table 3:** Vigor-productive variables (mean) of the ‘Gala Select’ (A) and ‘Fuji Suprema’ (B) on the Geneva® series rootstocks, under replanting area in Caxias do Sul municipality, southern Brazil.

Cultivar	Rootstock	Plant Height	TCSA^a^	Number of Branches	Canopy Volume	Productivity	Cumulative Yield	Productive Efficiency	Inc t_cm^2^**
**Gala Select**	G.202	2.96	8.38	21.35	1.30	37.88	113.64	1.70	1.00
	G.210	3.36	11.18	28.92	1.89	41.74	125.22	1.20	−0.26
	G.213	2.96	7.67	24.04	1.30	45.69	137.07	2.18	4.73
	G.814	3.49	11.88	27.68	1.66	41.16	123.47	1.21	−1.53
**Fuji Suprema**	G.202	3.30	11.24	23.66	2.94	29.70	89.11	0.73	4.00
	G.210	3.65	16.70	29.34	3.36	32.31	96.93	0.54	2.41
	G.213	2.85	7.33	20.37	1.49	32.03	96.10	1.30	6.35
	G.814	3.66	16.95	29.61	3.03	37.92	113.76	0.61	3.79

a Trunk cross-sectional area (cm^2^).

**Table 4:** Qualitative Fruit variables (mean) of the ‘Gala Select’ (A) and ‘Fuji Suprema’ (B) on the Geneva® series rootstocks, under replanting area in Caxias do Sul municipality, southern Brazil.

Cultivar	Rootstock	Firmness	Total Soluble Solids	CAT1^a^	Ripe Fruit	Caliber 100-120	Caliber 120-150	Caliber >150
**Gala Select**	G.202	66.71	13.85	99.38	100.00	1.19	42.68	57.38
	G.210	65.54	13.80	97.50	100.00	3.75	15.00	81.25
	G.213	71.06	15.24	100.00	100.00	2.44	14.76	82.80
	G.814	76.31	14.06	100.00	97.50	0.00	14.66	85.34
**Fuji Suprema**	G.202	78.71	15.52	92.50	52.50	16.25	38.75	45.00
	G.210	83.92	14.82	80.63	100.00	17.50	41.25	41.25
	G.213	76.77	16.38	88.75	85.00	10.00	46.25	43.75
	G.814	81.71	14.42	87.50	90.00	20.00	36.25	43.75

a Fruits had a red color of ≥50 %.

Despite the fact that G.213 is a dwarf rootstock, ‘Gala Select’/G.213 combination was the most productive combination during 2018/19, 2019/20, and 2020/21 growing seasons, with the highest cumulative yield performance and productive efficiency of 134 t ha^−1^ and 5.9 kg cm^−2^, respectively (Fig. 5A). ‘Gala Select’/G.213 was approximately 10 % and 14.5 % more productive than ‘Gala Select’/G.202 (117 t ha^−1^) and ‘Gala Select’/G.814 (121 t ha^−1^) and ‘Gala Select’/G.210 (123 t ha^−1^) (Fig. 5A).

The ‘Fuji Suprema’/G814 combination demonstrated the highest cumulative yield performance (115 t ha^−1^) during 2018/19, 2019/20, and 2020/21 growing seasons, despite that G.814 is a semi-dwarf rootstock (Fig. 5B). ‘Fuji Suprema’/G.814 combination was 34 % more productive than ‘Fuji Suprema’/G.202, ‘Fuji Suprema’/G.210, and ‘Fuji Suprema’/G.213 (Fig. 5B). Despite having the lowest cumulative yield performance, the ‘Fuji Suprema’/G.213 combination demonstrated the highest productive efficiency (3.8 kg cm^−2^) compared to other rootstocks (Fig. 5B).

Fig. 7 depicts the results of the PCA for fruit quality variables. According to fruit quality, the ‘Gala Select’ combinations under all rootstocks formed two distinct groups. ‘Gala Select’/G.213 and ‘Gala Select’/G.210 were the combinations that produced the greatest quantity and quality of fruits with the highest caliber (Fig. 7A). Thus, despite being the most productive, these combinations also produced fruits of the largest caliber and the same condition was observed in the ‘Fuji Suprema’ (Fig. 7B). The ‘Gala Select’ and ‘Fuji Suprema’/G.213 combination had the largest fruit caliber and the highest total soluble solids, and lesser firmness than the ‘Fuji Suprema’/G.202 and ‘Fuji Suprema’/G.814 combinations (Fig. 7).

‘Fuji Suprema’/G.213 and ‘Fuji Suprema’/G.202 combinations exhibited the highest total soluble solids and distinguished dwarfing from semi-dwarfing combinations. The ‘Fuji Suprema’/G.202 combination showed greater fruit firmness than the ‘Fuji Suprema’/G.213 combination. The ‘Fuji Suprema’/G.210 combination produced a greater quantity of larger fruits than the ‘Fuji Suprema’/G.814 combination, which produced fruits with greater firmness. The ‘Fuji Suprema’/G.213 demonstrated a strong positive correlation with classes of large and large and small fruits. Thus, ‘Fuji Suprema’/G.213 and ‘Fuji Suprema’/G.210 produced comparable quantities of large-sized fruit (Fig. 7B).

## 4 Discussion

The rootstocks vigor induced to the different combinations of the ‘Gala Select’ and ‘Fuji Suprema’ under the rootstocks G.202, G.814, G.210, and G.213 in both the Painel/SC and Caxias do Sul/RS regions, classified the G.814 and G.210 rootstocks as semi-dwarfing and the G.202 and G.213 rootstocks as dwarfing, corroborating with Petri [19], Denardi [4,5], Macedo [15], and Rufato [25]. The physio-morphological expression of the cultivar/rootstock combination is influenced by the soil, climate, rootstocks, and cultivar-canopy [29]. The vigorous rootstocks can be used in soils with low natural and/or shallow fertility, while the less vigorous rootstocks can be used in soils with high fertility, allowing the vegetative-productive balance of the cultivar-canopy to be maintained [17]. The ability to control the vigor of apple plants, thereby enabling denser plantings, was a primary objective in the development of the Geneva series rootstocks [24].

Differences in the cumulative productive performance (cumulative yield and productive efficiency) of ‘Gala Select’ and ‘Fuji Suprema’ combinations under G.202-G.213 (dwarfing) and G.210-G.814 (semi-dwarfing) rootstocks were observed for each vigor classification group, at the experimental site of Painel/SC region. During 2018/2019, 2019/2020, and 2020/2021 growing seasons, the ‘Gala Select’/G.210 (semi-dwarfing) combination and ‘Gala Select’/G.213 (dwarfing), produced a greater cumulative yield. However, the ‘Gala Select’/G.213 (dwarfing) combination demonstrated a 22 % higher productive efficiency than ‘Gala Select’/G.210 (semi-dwarfing), likely due to a greater capacity to control plant the vigor of the plants. The high cumulative yield of the ‘Gala Select’/G.210 was also observed by Reig [22] in an experiment that was developed in a replanting area in the United States of America (USA) using the ‘Super Chief Delicious’ cultivar.

The ‘Fuji Suprema’/G.814 (semi-dwarfing) combination had the highest cumulative yield over the evaluation period; however, the ‘Fuji Suprema’/G.213 (dwarfing) combination was 25 % more yield efficient (semi-dwarfing). The highest productive efficiency of dwarfing rootstocks (‘Fuji Suprema’/G.213) was observed in plants with regulated vegetative growth and the proper planting density [13]. Consequently, even under replanting conditions with two years of fallow, the evaluated Geneva rootstocks maintained the same vigor features that Denardi [5] and Macedo [15] observed in replanting areas. According to these authors, among dwarfing rootstocks, G.213 show the highest yield performance, regular production, productive efficiency, and average fruit mass. According to Macedo [15], the ‘Maxi Gala’/G.213 combination demonstrated greater precocity and productive efficiency in virgin soil and in replanting soil, as well as less alternate bearing in replanting soil, demonstrating the greater productive stability of the rootstock G.213. The precocity induced by the dwarfing rootstock is important for the return on the initial capital investment in the orchard [19]. This precocity permits densely planted crops with a high initial investment but a rapid financial return [13,14].

Similar to the Painel/SC region, differences in productive performance were observed between cultivars and rootstocks in the Caxias do Sul/RS region. The ‘Gala Select’/G.213 (dwarfing) combination produced a cumulative yield that was 21 % higher than ‘Gala Select’/G.202 combination and 70 % higher than ‘Gala Select’/G.814 and ‘Gala Select’/G.210 combinations, during the 2018/2019, 2019/2020 and 2020/2021 growing season. The ‘Fuji Suprema’/G.213 (dwarfing) combination showed a cumulative yield of 21 % higher than ‘Fuji Suprema’/G.202 combination and a 138 % higher productive efficiency than ‘Fuji Suprema’/G.814 combination over the same period of evaluation. Apple plants on the Geneva series rootstocks exhibit the highest productive performance in comparison to the traditional rootstocks used worldwide, such as M.9, M.26, and M.27 [8].

Due to the absence of thinning during the 2019/2020 growing season in order to maximize production, the yields of the ‘Gala Select’ under all evaluated Geneva rootstocks decreased in 2021. The average yield performance of these later combinations in the 2020 harvest was above the average of the previous year, at 76.85 t ha^−1^, reflecting a 59 % reduction in the ‘Gala Select’/G.202 (dwarfing) combination and ‘Gala Select’/G.814 (semi-dwarfing) and a 25 % reduction on ‘Gala Select’/G.213 (dwarfing) and ‘Gala Select’/G.210 (semi-dwarfing). Thus, the ‘Gala Select’/G.213 and ‘Gala Select’/G.210 combinations exhibited less alternate bearing. However, ‘Gala Select’/G.213 (dwarfing) combination productive efficiency was 80 % higher.

The ‘Gala Select’/G.213 and ‘Fuji Suprema’/G.213 (dwarfing) combinations had the best production stability over the evaluated years, and the fact these combinations were less susceptible to the alternate bearing when compared to the ‘Gala Select’/G.210 and ‘Fuji Suprema’/G.210 (semi-dwarfing) combinations, could be justified according to Reig [22], where rootstocks that are more efficient in controlling plants, vigor provided less apple alternate bearing. The alternate bearing is a result of the inhibition of the floral process brought on by the excessive production of gibberellins by apple seeds during their formation phase and the excessive consumption of plant reserves during years of high production [21,22].

Despite the difference in vigor, the ‘Fuji Suprema’/G.814 combination was approximately 21 % more productive than the other rootstocks in the Painel/SC and Caxias do Sul/RS regions, and there was no significant difference in average or cumulative yield. According to Lordan [13,14], the G.814 semi-dwarfing rootstock has high productivity and induces greater vigor to cultivate canopy than the apple stem grooving virus (ASGV) vulnerable M.9T337 rootstock [10].

‘Gala Select’ and ‘Fuji Suprema’/G.213 (dwarfing) combinations maintained greater productive efficiency than the same cultivars with other rootstocks, outperforming by 40 % the ‘Gala Select’ and ‘Fuji Suprema’/G.814 (semi-dwarfing) combinations in Painel/SC and Caxias do Sul/RS regions. The superior productive efficiency of ‘Gala Select’ and ‘Fuji Suprema’/G.213 (dwarfing) combinations resulted in a high average yield of five tones, which was attributable, in part, to an increase in trunk cross-sectional area (TCSA) of 35 % over G.814 (semi-dwarfing) and 52 % over G.210 (dwarfing). According to Petri [19], a high productive efficiency is a crucial characteristic that permits the densification of apple orchards, thereby enhancing yield performance.

‘Gala Select’ and ‘Fuji Suprema’ under G.213 (dwarfing) and G.814 (semi-dwarfing) rootstocks were the combinations with comparable fruit caliber (size), despite ‘Gala Select’ and ‘Fuji Suprema’/G.213 combinations presenting highly mature fruits at harvest and indicating harvest advance. According to Denardi [4] ‘Gala Select’/G.213 (semi-dwarfing) and ‘Fuji Suprema’/G.210 (dwarfing) combinations produced a greater quantity of large-sized fruit. However, Pasa [17] evaluated the fruit size in the ‘Fuji Suprema’ for various rootstocks and concluded that differences in fruit weight are not consistent over time and are likely not attributable to rootstock influence.

## 5 Conclusions

5.1. Geneva series rootstocks are well adapted under fallow land and replanting conditions in southern Brazil.

5.2. ‘Gala Select’ and ‘Fuji Suprema’ grafted under different Geneva series rootstocks are separate into two vigor groups, ‘Gala Select’ and ‘Fuji Suprema’/G.202 and G.213 (dwarfing) and ‘Gala Select’ and ‘Fuji Suprema’/G.210 and G.814 (semi-dwarfing).

5.3. The combinations ‘Gala Select’/G.213 (dwarfing) and ‘Gala Select’/G.210 (semi-dwarfing) are similar in yield performance, despite that the ‘Gala Select’ and ‘Fuji Suprema’/G.213 (dwarfing) combinations are superior in productive efficiency.

5.4. ‘Gala Select’/G.210 and G.213 and ‘Fuji Suprema’/G.210 and G.213 are the combinations with the largest fruit caliber in both Painel/SC and Caxias do Sul/RS regions, despite that ‘Gala Select’ and ‘Fuji Suprema’/G.210 combinations produce more amount of bigger fruit caliber.

5.5. ‘Gala Select’ and ‘Fuji Suprema’/G.213 combinations anticipate the fruit ripping with a higher degree of brix and a higher iodine-starch index in both Painel/SC and Caxias do Sul/RS regions.

5.6. ‘Gala Select’/G.210 (semi-dwarfing) and G.213 (dwarfing) are the combinations with high yield performance, productive efficiency and fruit quality.

5.7. ‘Gala Select’/G.213 (dwarfing) combination present a constant and better yield and is more reliable to the producer, and less vigorous, resulting in lower labor costs than ‘Gala Select’/G.210 combination.

## Funding

This study was supported by the Santa Catarina State University-10.13039/501100008128UDESC, the 10.13039/501100002322Coordination for the Improvement of Higher Education Personnel (CAPES), the 10.13039/501100003593National Council for Scientific and Technological Development (CNPq), and the Foundation for Support of Research and Innovation of the State of Santa Catarina (10.13039/501100005667FAPESC).

## Data availability statement

Data will be made available on request.

## Additional information

No additional information is available for this paper.

## CRediT authorship contribution statement

**Pricila Santos da Silva:** Investigation. **Juliana Martins de Lima:** Investigation. **Marllon Fernando Soares dos Santos:** Investigation. **Daiana Petry:** Methodology, Formal analysis. **Leo Rufato:** Supervision, Project administration, Methodology, Formal analysis, Conceptualization. **Francine Regianini Nerbass:** Visualization, Validation, Methodology. **Amauri Bogo:** Writing – review & editing, Writing – original draft, Funding acquisition, Conceptualization.

## Declaration of competing interest

On behalf of all Co-authors and been the Corresponding Author, I declare must disclose any financial and personal relationships with other people or organizations that could inappropriately influence (bias) their work.
